# Gut microbiome dysbiosis, short-chain fatty acid depletion and implications for neuroinflammation in atypical parkinsonian syndromes – a systematic review of patient cohorts

**DOI:** 10.1177/1877718X261485793

**Published:** 2026-09-17

**Authors:** Samiullah Amin Khan, Tobias Hegelmaier, Florian Wegner, Matthias Höllerhage, Melissa Peters, Alexander Duscha, Christiane Desel, Aiden Haghikia, Martin Klietz

**Affiliations:** 1 Department of Neurology, 9177 Hannover Medical School, Hannover, Germany

**Keywords:** progressive supranuclear palsy, multiple system atrophy, corticobasal syndrome, atypical parkinsonian syndromes, gut microbiome, dysbiosis

## Abstract

**Introduction:**

Atypical parkinsonian syndromes (APS) such as progressive supranuclear palsy (PSP), multiple system atrophy (MSA) and corticobasal syndrome (CBS) pose a major clinical challenge due to their progressive disease course, reduced life expectancy and resistance to dopaminergic therapy. The gut microbiome has gained increasing attention as a potential contributor to APS etiology and progression.

**Aim:**

The literature analysis aims to systematically evaluate alterations in the gut microbiome in APS, their correlation with clinical symptoms and their potential diagnostic and therapeutic relevance.

**Material and methods:**

PubMed and Web of Science were searched separately for ‘’progressive supranuclear palsy’’, ‘’multiple system atrophy’’ and ‘’corticobasal syndrome’’, each combined with “gut microbiome” AND “human”. Inclusion criteria were ≥ 10 subjects per group, clinically confirmed APS and gut microbiome analysis in a case-control, cross-sectional, longitudinal or randomized controlled trial study design. 7 studies met the inclusion criteria (1 PSP, 1 PSP/MSA, 5 MSA). No studies with CBS patients could be included.

**Results:**

The gut microbiome is altered in APS compared to healthy controls, with several alterations correlating with clinical symptoms in individual studies. A reduction of short-chain fatty acid (SCFA) producing bacteria and an enrichment of pro-inflammatory taxa were observed, alongside impaired markers of intestinal barrier integrity.

**Discussion and conclusion:**

APS are associated with dysbiosis that correlates with clinical symptoms, although causality remains to be established. Generalizability is limited due to the lack of geographic heterogeneity in the included studies. Longitudinal studies with larger cohorts are required to identify robust microbial markers and therapeutic potential.

## Plain language summary Is the gut microbiome altered in patients with atypical parkinsonian syndromes? A systematic review

Atypical parkinsonian syndromes, such as progressive supranuclear palsy, multiple system atrophy and corticobasal syndrome, are rare diseases similar to Parkinson's disease. However, people with atypical parkinsonian syndromes experience a faster worsening of physical and mental abilities and a shorter life expectancy than people with Parkinson's disease. The gut microbiome, the bacteria, viruses and fungi in our gut, has been linked to progressive neurological diseases. This raises the question of whether the gut microbiome is also altered in atypical parkinsonian syndromes. We systematically searched the scientific databases PubMed and Web of Science for studies on this topic, selected eligible studies based on predefined criteria and analyzed their findings. We found that the gut microbiome in patients with atypical parkinsonian syndromes is changed compared to healthy people. The main findings include an imbalance of the gut bacteria and a reduction in bacteria producing short-chain fatty acids. We also found signs of a weakened gut wall, which usually prevents harmful substances from leaking into the bloodstream. However, no studies with people with corticobasal syndrome were found. The findings suggest that patients with atypical parkinsonian syndromes have an altered gut microbiome that is partially linked to their symptoms. However, it is unclear whether these changes in the gut microbiome are causing the disease or are a consequence of it. Most of the studies were conducted in Asia, which means that the results may not be applicable to other groups of patients in the world because the composition of the gut microbiome differs regionally. In the future, larger studies with more geographically diverse patient groups are needed to confirm these results and to explore whether the gut microbiome could become a target for treatment options.

## Introduction

The most common atypical parkinsonian syndromes (APS) are progressive supranuclear palsy (PSP), multiple system atrophy (MSA) and corticobasal syndrome (CBS).^1^ In comparison to Parkinson's disease (PD), APS follow a more rapidly progressive disease course, leading to severe disability and a markedly reduced life expectancy of six to nine years from symptom onset.^2–4^ Furthermore, APS are largely resistant to dopaminergic therapy.^5^

PSP is the most common APS and is neuropathologically defined by the accumulation of 4R-tau protein, predominantly in the globus pallidus, subthalamic nucleus and substantia nigra.^6^ Clinically, PSP is characterized by early postural instability, oculomotor dysfunction, akinesia and cognitive impairment, with several recognized phenotypic subtypes such as Richardson's syndrome (PSP-RS).^7^ Gastrointestinal symptoms such as constipation and dysphagia are also frequently observed.^8^

CBS is a rare 4R-tauopathy that typically presents after the age of 50.^9^ Clinically, CBS manifests with asymmetric limb rigidity, apraxia, cortical sensory loss, myoclonus and the alien limb phenomenon, alongside deficits in executive and cognitive functions.^10^

MSA is a synucleinopathy characterized by the accumulation of alpha-synuclein in oligodendroglial cytoplasmic inclusions, leading to neurodegeneration in the basal ganglia, cerebellum and the autonomic nervous system.^11,12^ Two clinical subtypes are distinguished: MSA-P, dominated by parkinsonism and MSA-C, dominated by symptoms of cerebellar ataxia.^13^ Early autonomic features such as erectile dysfunction, orthostatic hypotension and urinary dysfunction are characteristic for MSA.^14^

The human body maintains a symbiotic relationship with approximately 38 trillion microorganisms,^15^ collectively termed microbiota.^16^ While the term microbiota refers to the assembly of microorganisms with their inherent features such as structure, metabolites and their genome, the microbiome encompasses the entire microbial community within a well-defined habitat characterized by distinct physiochemical characteristics forming a specific ecological niche.^16^ Important functions of the gut microbiome include the modulation of its environment to strengthen the host against exogenous pathogens,^17^ modulation of the immune system,^18^ stimulation of angiogenesis^19^ and the regulation of the brain via the gut-brain axis.^17^ Gut microbiome alterations have increasingly been described in neurodegenerative diseases,^20^ raising the question of whether similar changes may be present in APS. The aim of this systematic review is to examine alterations of the gut microbiome in atypical parkinsonian syndromes and to evaluate their clinical correlations.

## Material and methods

Data collection for the evaluation of relevant literature was conducted via the meta-databases PubMed and Web of Science according to predefined inclusion and exclusion criteria. The main research question was defined as: ‘’Does the gut microbiome in patients differ from healthy controls and do alterations of the gut microbiome correlate with clinical symptoms?’’. Studies had to include patients with a confirmed diagnosis of an APS such as MSA, PSP or CBS in whom the gut microbiome was analyzed via stool, with a minimum of 10 patients per cohort. Eligible study designs included case-control, cross-sectional and longitudinal observational studies as well as randomized controlled trials evaluating microbiome-targeted interventions. Studies without microbiome analysis, lacking clinical data, systematic reviews, meta-analyses and animal model studies were excluded. Only articles published up to December 1^st^, 2025, were considered.

**Figure 1.**
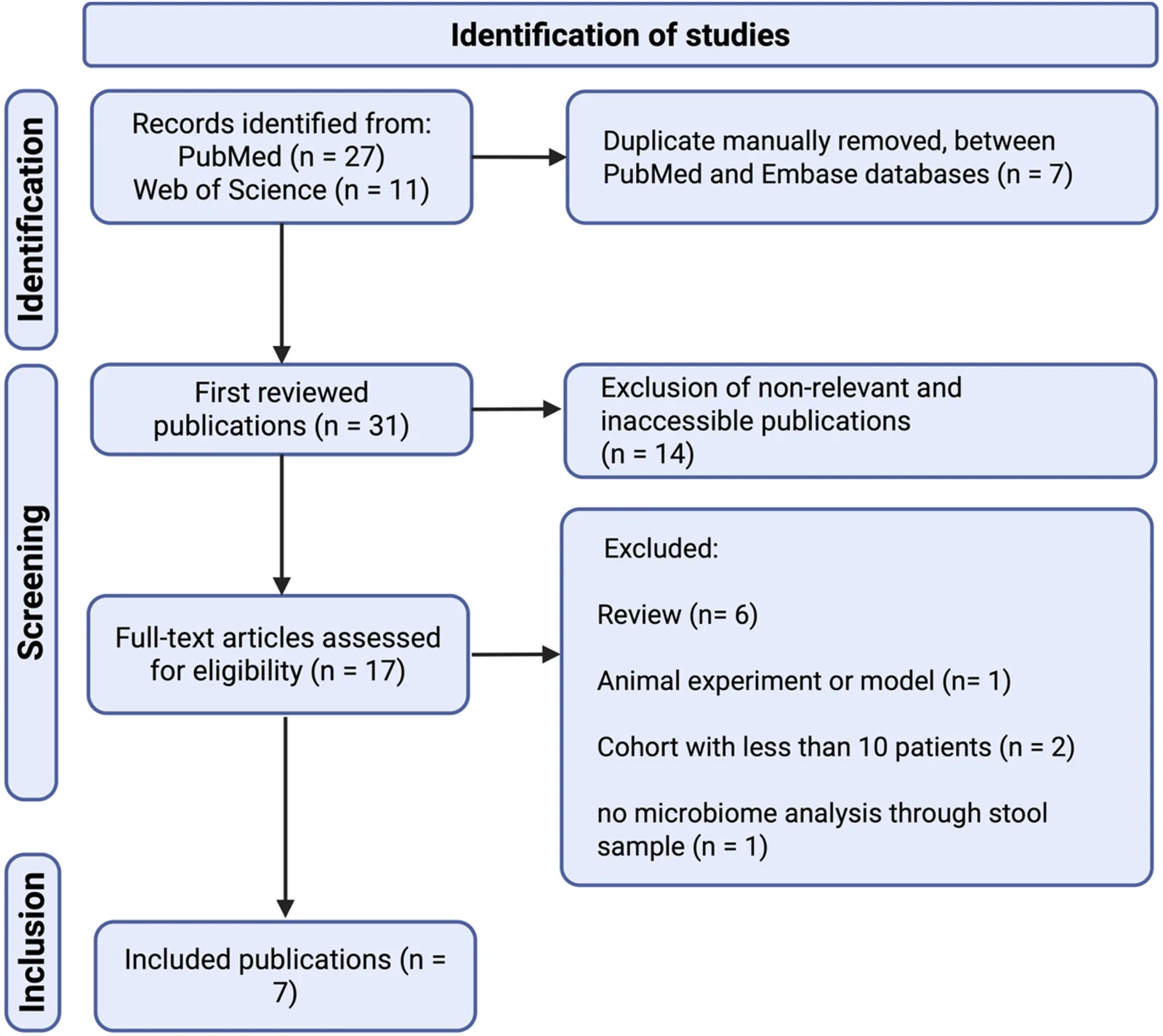
PRISMA flowchart of the process of literature research.

Three separate searches were conducted in each database with the following combinations: (1) ‘’progressive supranuclear palsy’’ AND ‘’gut microbiome’’ AND ‘’human’’ (2) ‘’multiple system atrophy’’ AND ‘’gut microbiome’’ AND ‘’human’’ (3) ‘’corticobasal syndrome’’ AND ‘’gut microbiome’’ AND ‘’human’’. No field restrictions were applied. All searches were performed on the meta-databases PubMed and Web of Science. The PubMed search was conducted on December 1, 2025, and the Web of Science search on July 17, 2026. First, all results were collected and duplicates were removed. Subsequently, studies were independently screened by two reviewers by title and abstract regarding the research question and the predefined inclusion and exclusion criteria. Studies deemed suitable during this process were then independently assessed in full-text form by both reviewers. Only the studies meeting all the criteria were ultimately included in this systematic review (n = 7). This process is summarized in the PRISMA flowchart (Figure 1). The systematic review was conducted in accordance with the PRISMA guidelines for systematic reviews. This review was not prospectively registered in PROSPERO. The included studies were assessed for risk of bias using design-matched tools: QUADAS-2 for studies reporting diagnostic accuracy measures (Supplementary Table 1),^21^ the Newcastle-Ottawa Scale for case-control studies (Supplementary Table 2)^22^ and the Cochrane Risk of Bias 2.0 tool for the randomized controlled trial (Supplementary Table 3).^23^ A quantitative meta-analysis was not performed given the substantial heterogeneity in study designs, sequencing platforms and bioinformatics pipelines as well as variability in dietary background across cohorts.

## Results

Overall, seven publications met the inclusion criteria and were included in the data analysis of this systematic review. Each of the included studies addressed both APS and the gut microbiome.

### Alterations of the gut microbiome in patients with PSP

Regarding alterations of the gut microbiome in patients with PSP, one randomized controlled trial (RCT)^24^ and data on PSP patients from an Italian multi-cohort study^25^ were included. The most important results are summarized in Table 1.

**Table 1.** Studies on changes in the gut microbiome in patients with PSP.

Study	Region	Participants	Methods	Results and discussion
Tian et al. (2023)	China	n = 118 (PSP-RS recently diagnosed (n = 68), HC (n = 50))	V3-V4 16S-rRNA sequencing	– Reduced abundance of several bacterial species (Firmicutes, *Blautia*, *Faecalibacterium*, *Roseburia*, *Agathobacter*, *Romboutsia*) – Increased abundance of Proteobacteria, *Lactobacillus* and *Klebsiella* – Increased alpha-diversity – Increased inflammatory markers (calprotectin, lactoferrin, α-1-antitrypsin, zonulin (in stool and serum)) (p < 0.0001) – Decreased expression of claudin-5, occludin, E-cadherin, ß-catenin (p < 0.0001) – Phospho-tau-accumulation in 74% of PSP-RS patients vs. 8% in HC (p < 0.0001) – Correlations: PSPRS score positively correlated with *Escherichia-Shigella*, *Lactobacillus* and *Klebsiella* (p < 0.05); motor subscales positively correlated with *Escherichia-Shigella* and *Klebsiella* (p < 0.05)
Barichella et al. (2019)	Italy	n = 350 (PD (n = 193), MSA (n = 22), PSP (n = 22), healthy controls (n = 113))	V3-V4 16S- rRNA sequencing	– Reduced abundance of *Lachnospiraceae* (p = 0.042) and its genus *Roseburia* (p = 0.003) – Increased abundances of certain *Ruminococcaceae* genera – Similar phylogenetic profiles of PSP and MSA; clear differences between APS and both PD and HC

This table describes the main findings of the studies on changes in the gut microbiome in PSP patients.

Abbreviations: APS: Atypical parkinsonian syndromes; HC: healthy controls; MSA: Multiple system atrophy; PD: Parkinson's disease; PSP: Progressive Supranuclear Palsy.

The phase 2 RCT by Tian et al. (2023) enrolled 68 newly diagnosed PSP-Richardson syndrome (PSP-RS) patients, who were randomized to receive either fecal microbiota transplantation (FMT) from healthy donors (n = 34) or a placebo solution of 0.9% saline and colorant (n = 34). An additional 50 spousal partners of enrolled patients were included as healthy controls (HC). Stool samples were analyzed by V3-V4 16S rRNA sequencing.

The baseline findings comparing the PSP-RS cohort with HC showed that alpha diversity was significantly increased in the PSP-RS group compared to HC. Taxonomically, at the phylum level, Proteobacteria were more abundant and Firmicutes were less abundant in PSP-RS patients. At the genus level, *Lactobacillus* and *Klebsiella* were significantly increased, while five genera (*Blautia, Faecalibacterium, Roseburia, Agathobacter* and *Romboutsia*) were significantly decreased in PSP-RS patients. Concentrations of the fecal markers calprotectin (p < 0.0001), lactoferrin (p < 0.0001), alpha-1-antitrypsin (p < 0.0001) and zonulin (p < 0.0001) were significantly increased in PSP-RS patients compared to HC. Serum zonulin levels were similarly elevated in the PSP-RS group. Immunohistochemical stainings of tissue biopsies obtained during colonoscopy revealed a decreased expression of the tight junction proteins claudin-5 and occludin in patients with PSP-RS compared to HC (p < 0.0001). Concurrently, the expression of the adherens junction proteins E-cadherin and ß-catenin were significantly reduced in patients with PSP-RS. Furthermore, an accumulation of phosphorylated tau in the colonic mucosa was detected in 74% of PSP-RS patients, whereas the proportion in HC was 8% (p < 0.0001). The enrichment of the genera *Escherichia-Shigella*, *Lactobacillus* and *Klebsiella* positively correlated with the PSP rating scale (PSPRS). The motor subscale score PSPRS-V + VI was also positively correlated with a higher abundance of *Escherichia-Shigella* and *Klebsiella*.^24^

In another study (n = 350), Barichella et al. (2019) investigated the gut microbiome in 22 PSP patients, 193 PD patients, 113 HC and 22 MSA patients. Alpha diversity showed no significant differences in PSP patients compared to HC, which was in contrast to PD patients. A reduction in *Lachnospiraceae* (p = 0.042), including its genus *Roseburia* (p = 0.003), was observed in patients with PSP compared to HC. Additionally, certain genera within *Ruminococcaceae* showed higher abundances in patients with PSP compared to HC. Phylogenetically, the microbiome composition of HC and PD patients differed significantly from that of APS (PSP and MSA), which were phylogenetically similar to each other.^25^

### Alterations of the gut microbiome in patients with MSA

The following section addresses gut microbiome alterations in MSA patient cohorts. Table 2 provides an overview of the results.

**Table 2.** Studies on changes in the gut microbiome in patients with MSA.

Study	Region	Participants	Methods	Results and discussion
Tan et al. (2018)	Malaysia	n = 34 (MSA (n = 17), HC (n = 17))	V3-V4 16S-rRNA sequencing	– Significantly reduced abundance of *Paraprevotella* (p < 0.001) – Significantly increased abundance of *Bacteroides* (particularly *B. fragilis*) (p = 0.003) – Reduced levels of SCFAs, choline and trimethylamine – Constipation significantly correlates with dysbiosis on univariate analysis (p < 0.05) – No significant correlation with BMI, constipation or levodopa on multivariate analysis
Barichella et al. (2019)	Italy	n = 350 (PD (n = 193), MSA (n = 22), PSP (n = 22), HC (n = 113))	V3-V4 16S-rRNA sequencing	– Reduced abundance of *Prevotellaceae* and *Faecalibacterium* – Increased abundance of *Lactobacillaceae* – Altered composition of *Ruminococcaceae*
Du et al. (2019)	China	n = 80 (MSA (n = 40), HC (n = 40))	V3-V4 16S-rRNA sequencing	– Increased abundance of *Lactobacillus*, *Gordonibacter* and *Phascolarctobacterium* – Reduced abundance of *Haemophilus* (stool) and *Leucobacter* (blood) – Increased abundance of *Bacteroides* (blood) – Alpha diversity unchanged; beta diversity significantly altered – Negative correlation of *Haemophilus* with disease duration, depression and anxiety – Positive correlation of *Gordonibacter* with SCOPA-AUT score
Du et al. (2024)	China	n = 146 (MSA (n = 42), PD (n = 40), iRBD (n = 29), HC (n = 35))	V3-V4 16S-rRNA sequencing	– Reduced SCFA levels in iRBD, MSA and PD – SCFA reduction correlates with diminished abundance of SCFA-producing bacteria – Negative correlation of propionate with Wexner score (p = 0.007) and HAMD (p = 0.029) – Negative correlation of acetate with autonomic symptoms – Differentiation based on biomarker profiles: AUC 0.809 (iRBD), 0.794 (MSA) and 0.701 (PD)
Wan et al. (2019)	China	n = 30 (MSA (n = 15), HC (n = 15))	Shotgun metagenomic sequencing	– Increased abundance of Verrucomicrobia, *Akkermansia*, *A. muciniphila* and *Roseburia hominis* – Decreased abundance of Actinobacteria, *Bifidobacterium*, *Blautia*, *Megamonas*, *Aggregatibacter*, *B. coprocola* and *B. pseudocatenulatum* – Reduced galactose, methane, pantothenate and CoA biosynthesis pathways
Liu et al. (2025)	Taiwan	n = 119 (MSA (n = 26), PD (n = 66), HC (n = 27))	V3-V4 16S-rRNA sequencing	– Decreased abundance of *Fusicatenibacter* – Interaction between *Ruminococcus gnavus* and *Erysipelatoclostridium* – Increased arginine degradation (q = 0.003) – Decreased PWY-6478 biosynthesis (q = 0.015)

This table focuses on the studies on changes in the gut microbiome in the studies with MSA cohorts.

Abbreviations: AUC: Area under the curve; *B*: *Bacteroides*; HC: healthy controls; MSA: multiple system atrophy; PD: Parkinson's disease; SCFA: short chain fatty acid; SCOPA-AUT: Scale for Outcomes in Parkinson's Disease for Autonomic Symptoms.

In a Malaysian cohort, Tan et al. (2018) analyzed fecal samples of 17 patients with MSA and 17 age-matched spouses or siblings from the same household environment using 16S rRNA sequencing of the V3-V4 region. At the operational taxonomic unit (OTU) and genus levels, a 5-fold reduction of *Paraprevotella* was found in patients with MSA compared to HC (adjusted p < 0.001). Furthermore, a higher abundance of the genus *Bacteroides* (particularly *Bacteroides fragilis*) was observed (adjusted p = 0.003). Significantly lower fecal levels of SCFAs, choline and trimethylamine were detected in the MSA cohort. The authors identified that the severity of constipation was associated with dysbiosis. Overall, significantly worse constipation levels were noted in MSA patients. No associations were found between gut microbiome alterations and confounders such as body mass index (BMI), constipation or levodopa dose.^26^

Barichella et al. (2019) also investigated the gut microbiome in 22 patients with MSA (n = 350, see also PSP section 3.1). Unlike in PD and PSP, there was no significant reduction in *Lachnospiraceae* in MSA patients. In the MSA cohort, *Prevotellaceae* and *Faecalibacterium* were less abundant, and the composition of the *Ruminococcaceae* genera was altered. Specifically in MSA patients, an increased abundance of *Lactobacillaceae* was observed.^25^

In another study (n = 80), Du et al. (2019) used 16S rRNA sequencing of the V3-V4 region to examine both the fecal microbiome and cell-free microbial DNA in blood samples from 40 MSA patients and 40 HC. Alpha diversity showed no significant differences between the MSA and HC cohorts. Regarding the beta diversity, no significant difference was observed between the two groups based on weighted UniFrac, while unweighted UniFrac revealed a significant difference between MSA and HC. In fecal samples, the genera *Lactobacillus*, *Gordonibacter* and *Phascolarctobacterium* were more abundant, while *Haemophilus* was less abundant in the MSA cohort compared to HC. Circulating microbial signatures in blood indicated increased relative abundance of *Bacteroides* and decreased relative abundance of *Leucobacter*. *Haemophilus* was negatively correlated with disease duration, depression and anxiety. The Scales for Outcomes in Parkinson's Disease - Autonomic Dysfunction (SCOPA-AUT) were positively correlated with the abundance of *Gordonibacter*.^27^

Du et al. (2024) also investigated the gut microbiome in patients with idiopathic REM sleep behavior disorder (iRBD, n = 29), MSA (n = 42), PD (n = 40) and HC (n = 35) using V3-V4 16S rRNA sequencing. Significantly lower fecal levels of SCFAs were detected in the iRBD, MSA and PD cohorts compared to HC, correlating with a decline in SCFA-producing bacteria. Propionic acid was negatively associated with the Wexner Constipation Score (p = 0.007) and the Hamilton Depression Rating Scale (HAMD) (p = 0.029). Acetic acid correlated negatively with autonomic symptoms (p = 0.043). In this study, ROC curve analysis demonstrated that the SCFA combination of propionic, acetic and butyric acid showed varying diagnostic accuracy in distinguishing patients with synucleinopathies from HC, with the highest discriminatory capacity for iRBD (AUC = 0.809), followed by MSA (AUC = 0.794), while the AUC for PD was comparatively lower (AUC = 0.701).^28^

Wan et al. (2019) investigated fecal samples using shotgun metagenomic sequencing in a cohort of 15 patients with MSA and 15 HC. The researchers identified a significant dysbiosis in MSA patients. At the phylum level, Verrucomicrobia were increased, while Actinobacteria were significantly decreased. At the genus level, this was reflected by a decreased abundance of *Bifidobacterium*, *Blautia*, *Megamonas* and *Aggregatibacter*, alongside a higher abundance of *Akkermansia*. At the species level, *Akkermansia muciniphila* and *Roseburia* hominis were more abundant, with a concurrent reduction in *Bacteroides coprocola* and *Bifidobacterium pseudocatenulatum*. Functional analysis via KEGG revealed reduced metabolic pathways concerning galactose, methane, pantothenic acid and CoA biosynthesis.^29^

Liu et al. (2025) compared the gut microbiome of MSA (n = 26) and PD patients (n = 66) with HC (n = 27). Significantly reduced levels of *Fusicatenibacter* were observed in patients with MSA compared to HC, however no significant difference was found between MSA and PD. Furthermore, the MSA cohort uniquely exhibited notable positive interbacterial co-abundance between the *Ruminococcus gnavus* group and *Erysipelatoclostridium*. Patients with MSA also showed significantly increased arginine degradation (q = 0.003) and decreased biosynthesis in the PWY-6478 pathway (GDP-D-glycero-alpha-D-mannoheptose biosynthesis) (q = 0.015) compared to HC, while no significant pathway differences were identified between MSA and PD. No correlation was found between the reduction of *Fusicatenibacter* in MSA and constipation. Similarly, constipation had no significant impact on alpha or beta diversity of the microbiome. A Random Forest classifier was able to distinguish MSA from PD patients based on microbial features (AUC = 0.78). Laxative use was elevated in both patient groups (MSA: 50%, PD: 50%, HC: 7.4%, p = 0.001). Probiotic use exhibited similar patterns (MSA / PD: 50%, HC: 7.4%, p = 0.001). MSA patients had a shorter disease duration (2.0 ± 1.3 years) compared to PD patients (4.1 ± 4.6 years; p = 0.004).^30^

## Discussion

**Figure 2.**
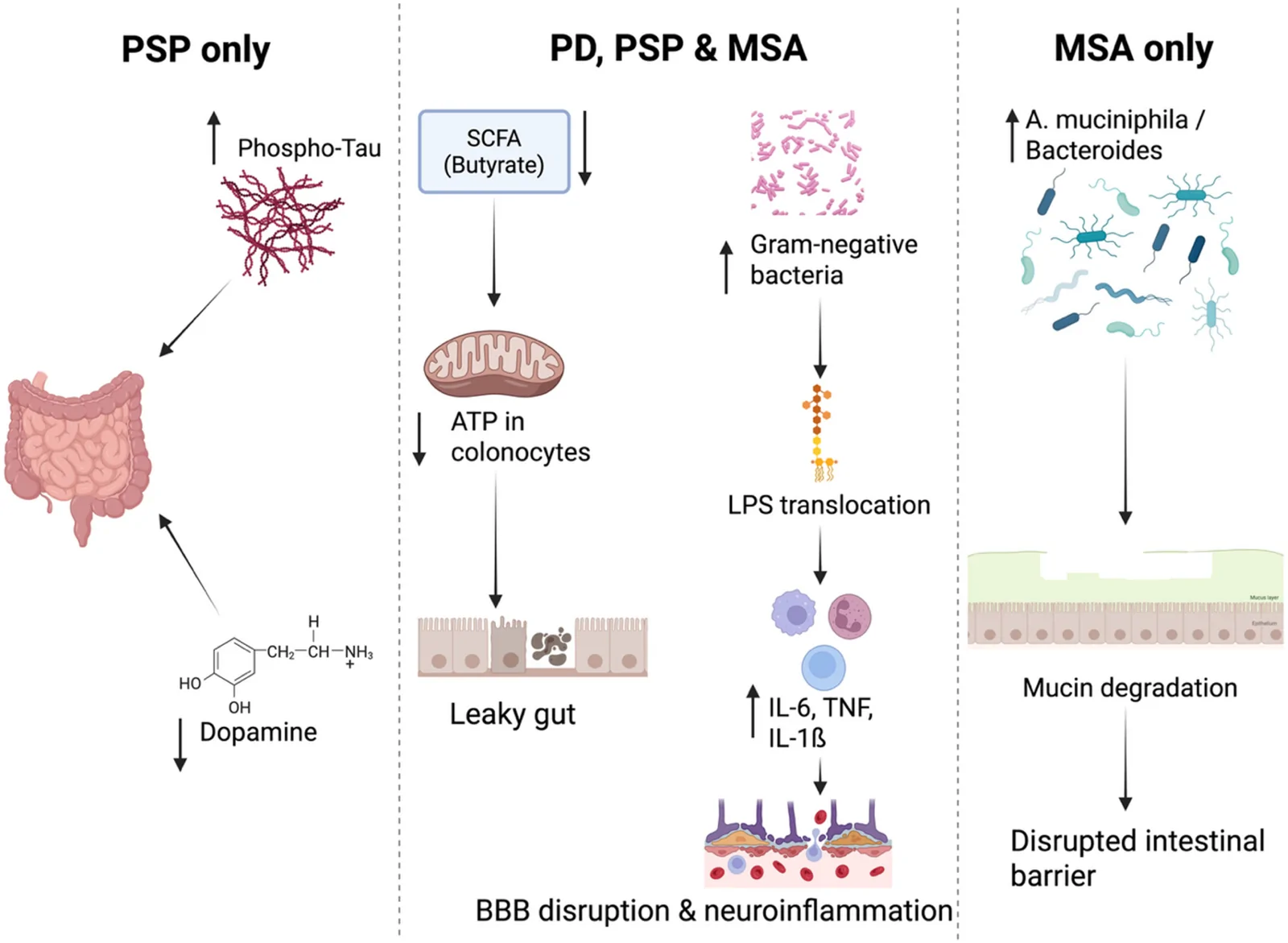
Possible influences of gut dysbiosis on atypical parkinsonian syndromes

The results of this systematic review suggest that the gut microbiome in APS is characterized by a depletion of SCFA-producing bacteria and dysfunction of the intestinal barrier. Since no CBS findings could be included, the following findings therefore solely concern MSA and PSP. It also has to be acknowledged that PSP and MSA are two biologically different entities, PSP is a 4R-tauopathy^6^ and MSA a synucleinopathy.^11^ Nonetheless, the potential involvement of the gut microbiome may be similarly relevant to both diseases. The included studies suggest that specific taxonomic profiles may allow differentiation between APS patients and HC. Collectively, multiple studies indicate that the gut microbiome represents a relevant factor in the pathophysiology of APS, with potential diagnostic and therapeutic implications for APS. Figure 2 visualizes the most important findings of the studies.

schematic overview of proposed intestinal pathomechanisms in parkinsonian syndromes. The central panel illustrates mechanisms potentially common to PD, PSP and MSA: reduced SCFA-production including butyrate deficiency, resulting in lack of energy of colonocytes and compromising intestinal barrier integrity (leaky gut).

The left panel depicts PSP-associated findings, including reduced dopamine levels and elevated phospho-tau protein in the gut.

The right panel illustrates specific alterations found in MSA; characterized by an increased abundance of mucin degrading bacteria (A. muciniphila or Bacteroides) causing disruption of the intestinal barrier.

Abbreviations: A. muciniphila: Akkermansia muciniphila; BBB: blood-brain barrier; LPS: lipopolysaccharides; MSA: multiple system atrophy; PD: Parkinson's disease; PSP: progressive supranuclear palsy; SCFA: short-chain fatty acids.

### General dysbiosis in APS

A predominant finding across most studies in this review on APS is the reduced abundance of SCFA-producing bacteria^24–26,28–30.^ SCFAs – primarily butyrate, propionate and acetate – are produced in the gut as end products of the anaerobic fermentation of resistant carbohydrates by intestinal microbiota.^31^ SCFA-producing bacteria were defined as taxa established in the literature as major producers of these metabolites, including *Lachnospiraceae*, *Roseburia*, *Faecalibacterium* and *Blautia*^32^ as well as *Fusicatenibacter.*^33^ As early as 1980, it was demonstrated in human tissue preparations that colonocytes of the intestinal epithelium use the SCFA butyrate as their primary source of energy, covering over 70% of their energy requirements.^34^ Colonocytes primarily maintain tight junctions,^35^ mucin production^36^ and active immune defense through the release of antimicrobial peptides^37^ as well as the cytokine IL-18.^38^ In a mouse-model, butyrate deficiency led to significant energy deprivation in colonocytes, resulting in increased tight junction permeability and the development of a leaky gut.^39^ These findings are consistent with those of Tian et al. (2023), who observed significantly reduced tight junction proteins and elevated zonulin concentrations in stool and serum samples.^24^ Collectively, these findings suggest that the reduced abundance of SCFA producing bacteria may contribute to impaired intestinal barrier integrity in APS, although direct mechanistic evidence in human cohorts remains limited. Additionally, since the included studies are cross-sectional, it is unclear whether SCFA depletion is a cause or consequence of APS.

Four out of the seven included studies showed increased abundances of Gram-negative bacteria in patients with APS.^24,26,27,29^ Gram-negative bacteria release lipopolysaccharide (LPS), an endotoxin, from their outer membrane.^40^ The previously described impairment of intestinal barrier integrity may facilitate LPS translocation into the systemic circulation, where it activates peripheral monocytes and promotes pro-inflammatory cytokine production, including TNF-α and IL-1ß via the TLR4 signaling pathway.^40^ This pathomechanism represents a component of the endotoxin hypothesis for PD, formalized by Brown et al. (2023), which encompasses a broader cascade including α-synuclein aggregation in enteric neurons, vagal propagation to the brain, microglial activation and progressive loss of dopaminergic neurons in the substantia nigra.^41^ This cascade may also be of pathophysiological relevance to APS. However, direct evidence for the applicability of the endotoxin hypothesis to APS remains lacking, as the hypothesis was developed on the basis of PD-specific evidence, including experimental mouse models of PD and clinical data from PD cohorts.

Both PSP and MSA patients showed an overabundance of *Lactobacillaceae* or the genus *Lactobacillus* across multiple studies, which correlated with worsening clinical symptoms.^24,25^ In PSP, the abundance of *Lactobacillus* was associated with a higher PSPRS score.^24^ This is surprising, as the *Lactobacillaceae* family is generally regarded as probiotic and beneficial for health.^42^ This apparent paradox has previously been noted in PD and the review by Van der Maden et al. (2025) demonstrates considerable heterogeneity in the effects of species within the *Lactobacillaceae* family, arguing that the seemingly contradictory observations can be attributed to species-specific differences that cannot be resolved by hypervariable region-targeted rRNA sequencing^43^ as employed in the majority of the included studies. This underscores the need for future APS studies employing more sensitive sequencing methods such as shotgun metagenomic sequencing or full-length 16S rRNA sequencing, as well as multi-omics approaches integrating metagenomics, metabolomics and host transcriptomics to obtain deeper functional insights.^44^

### Dysbiosis in PSP

Regarding the PSP-specific dysbiosis, Tian et al. (2023) postulated that reduced intestinal dopamine concentrations may contribute to the pathophysiology of constipation in PSP.^24^ In a mouse model, the presence of dopamine receptors D1 through D5 in the gut was demonstrated, with functional analyses primarily focusing on the receptors D2 and D3. Only the D2 receptor was found to influence intestinal motility: it is suggested to inhibit the presynaptic release of acetylcholine from enteric neurons, thereby reducing motility and slowing peristalsis to facilitate nutrient absorption.^45^ However, in this mouse model, mice lacking the D2 receptor exhibited enhanced motility and impaired absorption, contradicting the findings of Tian et al. (2023). In their study, PSP-RS patients with lower intestinal dopamine concentrations exhibited reduced motility, which manifested clinically as constipation. The authors themselves acknowledge that reduced intestinal dopamine concentrations may be a contributing factor to PSP pathophysiology, however, they consider dysfunctional intestinal barrier integrity and inflammatory processes to be the primary pathophysiological mechanisms in PSP.^24^

Regarding the correlations reported by Tian et al. (2023), the heterogeneity of associations between bacterial genera and cognitive test scores is notable: while the MoCA negatively correlated with *Klebsiella* and positively with *Romboutsia*, the MMSE showed a negative correlation with *Eubacterium coprostanoligenes* and a positive correlation with *Blautia*.^24^ Given this heterogeneity, the question arises as to whether potential statistical bias may be present. It is unclear whether a correction for multiple comparisons was performed in this study, as this is not explicitly stated in the publication. In the absence of such adjustments, false-positive results cannot be excluded.^46^

The FMT resulted in a significant improvement in motor symptoms (4.3 points on the PSPRS), normalized intestinal dopamine concentrations, reduced gastrointestinal complaints with a dropout rate of 7%.^24^ However, confounders such as diet and concomitant medication were not controlled for. Since diet can substantially influence microbiome composition,^47^ differences in dietary habits may have led to confounding, a limitation that applies to the majority of the included studies. Furthermore, study participants received antibiotics (ciprofloxacin and metronidazole) in the five days preceding FMT, which can markedly alter microbiome composition.^48^ Future RCTs controlling for diet, medication and antibiotic pre-treatment are required to evaluate FMT as a therapeutic option.

### Dysbiosis in MSA

In five of the six studies including MSA patients, a reduction of SCFA-producing bacteria was observed.^25–27,29,30^ Du et al. 2019 represents the only exception, as no significant reduction of SCFA-producing bacteria was detected compared to HC.^27^ The discrepancy may have been caused by differences in recruited HC because most of the MSA studies used controls from hospital or community settings,^25,28–30^ while Du et al. 2019 and Tan et al. 2018 matched patients with cohabiting partners.^26,27^ People living in the same household have been shown to have similar compositions of the gut microbiome,^49^ therefore differences in HC cohorts may also have led to different results. However, other factors such as differences in cohort size, medication status could also have caused the discrepancy. The overall consistency of SCFA depletion across the remaining the studies with MSA cohorts nonetheless suggests that reduced SCFA-producing bacteria may constitute a robust feature of gut dysbiosis in MSA.

In two of the six studies with MSA cohorts, an increased abundance of mucin-degrading genera was observed.^26,29^ The mucous layer consists of two layers, with the inner layer serving to separate the microbiota from the epithelium and the outer layer being colonized by bacteria supplied with nutrients via O-glycans.^49^ Wan et al. (2019) identified an increased prevalence of *Akkermansia muciniphila*,^29^ which, according to biochemical analyses, is capable of enzymatically degrading the entire mucous layer.^50^ The role of *Akkermansia muciniphila* appears to be dependent on its colonization density: In inflammatory bowel disease, where *Akkermansia muciniphila* is markedly reduced, the supplementation of *Akkermansia muciniphila* has been associated with restored mucosal barrier function, decreased pro-inflammatory cytokine levels and enhanced regulatory t-cells.^51^ However, overabundance of *Akkermansia muciniphila* in mice models with colorectal cancer has been shown to create an imbalance between mucin degradation and goblet-cell mediated restoration, resulting in downregulation of tight junction proteins and disruption of intestinal barrier integrity.^52^ Therefore, the overabundance of *Akkermansia muciniphila* as observed in Wan et al. (2019) may lead to excessive mucin degradation, eliminating the separation between microbiota and the epithelium and potentially resulting in a disrupted intestinal barrier. A similar observation was made in Tan et al. (2018), in which an increased prevalence of *Bacteroides* was reported,^26^ a genus likewise capable of enzymatic mucin degradation.^53^ Interestingly, an elevated abundance of *Bacteroides* is generally associated with higher dietary intake of animal protein and fat,^54^ however Tan et al. (2018) observed increased *Bacteroides* prevalence in the MSA cohort despite lower consumption of animal protein and fat.^26^ As these studies are cross-sectional in design, causal inferences cannot be drawn, and this aspect warrants further investigation in longitudinal studies with direct assessment of intestinal barrier parameters such as tight junction proteins.

### Limitations

Several limitations of this systematic review have to be acknowledged, concerning both the design of the included studies and the methodology of this review itself. A central limitation of the included studies is their cross-sectional design, which precludes causal inferences regarding the relationship between gut dysbiosis and APS pathophysiology. Furthermore, the majority of the studies relied on target 16S rRNA sequencing, with substantial heterogeneity across sequencing platforms, DNA extraction protocols and bioinformatics pipelines, restricting taxonomic resolution to the genus level and limiting cross-study comparability. Variability in sample collection, storage and processing further compounds these methodological inconsistencies, potentially contributing to conflicting findings between the studies. While internal validity of the studies was assessed using design-matched risk-of-bias tools, a formal certainty of evidence assessment such as GRADE was not performed. Recurring findings such as reduced SCFA-producing bacteria or increased abundances of Gram-negative bacteria were based on different taxa in most studies rather than a single identical outcome. Therefore, the requirements for a GRADE assessment were not met.^55^ Additionally, the studies solely addressed bacteria of the gut microbiome, however, fungi and viruses may also play a role in the pathophysiology of APS. A further gap in the current research is the complete absence of studies investigating the gut microbiome in CBS, which precluded any conclusions regarding this condition in this review. The included cohorts lack geographic diversity, with six of the seven cohorts originating from Asian populations and only one European study meeting the inclusion criteria. Since the microbiome is substantially influenced by factors such as diet, environmental exposures and genetic background,^56^ the generalizability of the findings to other populations is limited. This constraint also limits the external validity of the FMT results reported in Tian et al. (2023). Studies with more geographically diverse cohorts are required to evaluate the efficacy and safety of FMT. Disease-related symptoms such as autonomic dysfunction, constipation or dysphagia leading to reduced food intake in PSP^8^ and MSA^12,57^ have to be taken into consideration as a limiting factor when interpreting the microbial changes in APS compared to HC. This is further complicated by the inconsistent adjustment for confounders such as diet, medication use and disease duration across studies.

## Conclusion

This systematic review examined the extent to which the gut microbiome is altered in APS and investigated clinical correlates of these changes in relation to clinical symptoms. The current research suggests that a significant dysbiosis is present in APS, which correlates with clinical manifestations of these conditions. Whether the dysbiosis is causally implicated in the pathophysiology of APS or arises as a consequence of the disease remains unclear. Therefore, future controlled longitudinal studies are highly warranted to establish causality, with the integration of multi-omics enabling a better understanding of underlying pathomechanisms.

## Supplemental Material

sj-docx-1-pkn-10.1177_1877718X261485793 - Supplemental material for Gut microbiome dysbiosis, short-chain fatty acid depletion and implications for neuroinflammation in atypical parkinsonian syndromes – a systematic review of patient cohorts Supplemental material, sj-docx-1-pkn-10.1177_1877718X261485793 for Gut microbiome dysbiosis, short-chain fatty acid depletion and implications for neuroinflammation in atypical parkinsonian syndromes – a systematic review of patient cohorts by Samiullah Amin Khan, Tobias Hegelmaier, Florian Wegner, Matthias Höllerhage, Melissa Peters, Alexander Duscha, Christiane Desel, Aiden Haghikia and Martin Klietz in Journal of Parkinson's Disease
