# Supplementary material for: Gut microbiome dysbiosis, short-chain fatty acid depletion and implications for neuroinflammation in atypical parkinsonian syndromes – a systematic review of patient cohorts
Source: J Parkinsons Dis. 2026 Sep 17:1877718X261485793. Online ahead of print. doi: 10.1177/1877718X261485793 (PMC13586076; doi:10.1177/1877718X261485793)
Supplement: Supplementary file 1 [file sj-docx-1-pkn-10.1177_1877718X261485793.docx]

**Supplementary Table 1: QUADAS-2 score**

| Study | Risk of Bias | | | | Concerns regarding applicability | | |
| --- | --- | --- | --- | --- | --- | --- | --- |
|  | Patients Selection | Index Test | Reference Standard | Flow and Timing | Patients Selection | Index Test | Reference Standard |
| Du et al. (2024) | ? | + | ? | ? | + | + | ? |
| Liu et al. (2025) | ? | + | + | + | + | + | + |

This table shows the risk of bias of the used studies using the QUADAS-2 score.

QUADAS-2: Quality Assessment of Diagnostic Accuracy Studies 2; +: low risk of bias/ low concern regarding applicability; ?: unclear risk of bias/ unclear concern regarding applicability

**Supplementary Table 2: Newcastle-Ottawa Scale**

| Study | Newcastle-Ottawa-Scale | | | Overall Risk of Bias |
| --- | --- | --- | --- | --- |
|  | Selection (Total of 4) | Comparability (Total 2) | Outcome / Exposure (Total 3) | (Total score) |
| Barichella et al. (2019) | *** | ** | ** | Low (7) |
| Tan et al. (2018) | *** | ** | ** | Low (7) |
| Du et al. (2019) | **** | ** | ** | Low (8) |
| Wan et al. (2019) | *** | ** | ** | Low (7) |

Score: 0-3: High Risk for Bias, 4-6: Moderate Risk for Bias, 7-9: Low Risk for Bias. * Good quality: 3 or 4 stars in selection domain AND 1 or 2 stars in comparability AND 3 or 4 stars in outcome / exposure domain. Fair quality: 2 stars in selection domain AND 1 or 2 stars in comparability domain AND 2 or 3 stars in outcome / exposure domain. Poor Quality: 0 or 1 stars

**Supplementary Table 3: Cochrane Risk of Bias 2.0**
